# Metformin Treatment Has No Beneficial Effect in a Dose-Response Survival Study in the SOD1^G93A^ Mouse Model of ALS and Is Harmful in Female Mice

**DOI:** 10.1371/journal.pone.0024189

**Published:** 2011-09-01

**Authors:** Hannah M. Kaneb, Paul S. Sharp, Nazanin Rahmani-Kondori, Dominic J. Wells

**Affiliations:** 1 Wolfson Neuroscience Laboratories, Faculty of Medicine, Imperial College, Hammersmith Hospital Campus, London, United Kingdom; 2 Department of Veterinary Basic Sciences, Royal Veterinary College, London, United Kingdom; 3 Academic Neurology Unit, Sheffield Institute for Translational Neuroscience, Sheffield University, Sheffield, United Kingdom; National Institutes of Health, United States of America

## Abstract

**Background:**

Amyotrophic Lateral Sclerosis (ALS) is a devastating neurological disorder characterized by selective degeneration of upper and lower motor neurons. The primary triggers for motor neuron degeneration are unknown but inflammation, oxidative stress and mitochondrial defects have been identified as potential contributing factors. Metformin is an anti-type II diabetes drug that has anti-inflammatory and anti-oxidant properties, can bring about mitochondrial biogenesis and has been shown to attenuate pathology in mouse models of Huntington's disease and multiple sclerosis. We therefore hypothesized that it might increase survival in the SOD1^G93A^ murine model of ALS.

**Methodology/Principal Findings:**

Treatment of male and female SOD1^G93A^ mice (n = ≥6 per sex) with 2 mg/ml metformin in the drinking water from 35 days, resulted in a significant increase in motor unit survival, as measured by *in vivo* electrophysiology at 100 days, in male EDL muscles (24+/−2 vs. 14+/−2 motor units, p<0.005) and female TA muscles (21+/−1 vs. 15+/−2 motor units, P = 0.0134). We therefore continued to test the effect of 0.5, 2 and 5 mg/ml metformin in the drinking water from 35 days on disease onset and progression (identified by twice weekly determination of weight and neurological score) as well as survival in male and female SOD1^G93A^ mice (n = ≥14 per sex). Results for all groups were compared using Kaplan-Meier time to event analyses. In this survival study, metformin was unable to reduce pathology at any dose and had an unexpected dose-dependent negative effect on the onset of neurological symptoms (P = 0.0236) and on disease progression (P = 0.0362) in female mice.

**Conclusions/Significance:**

This study suggests that metformin is a poor candidate for clinical trial in ALS patients and that the possibility of harmful effects of metformin in female ALS patients with type II diabetes should be investigated.

## Introduction

Amyotrophic Lateral Sclerosis (ALS [OMIM 105400]) is a fatal neurodegenerative disorder associated with the selective degeneration of upper and lower motor neurons, for which there are currently no effective therapeutics [1]. Although the majority of ALS cases are sporadic, approximately 10% have a familial origin and of these 15–20% are caused by gain of function mutations in the copper/zinc superoxide dismutase (SOD1) gene (OMIM 147450) [2]. The primary triggers for motor neuron degeneration in ALS remain elusive, however research in patients and mutant SOD1 based models has revealed several processes that are likely to contribute to pathology, including but not limited to: inflammation and toxic glial activation [3], [4], mitochondrial dysfunction [5] and oxidative stress[3], [5].

Metformin is a small molecule activator of the metabolic regulator, AMP-activated protein kinase (AMPK) (OMIM numbers: α1 subunit 602739, α2 subunit 600497, β1 subunit 602740, β2 subunit 602741, γ1 subunit 602742, γ2 subunit 602743), which is routinely used for the treatment of type II diabetes [6], [7]. Metformin was originally prescribed for diabetes on the basis of its ability to reduce hepatic glucose production and increase insulin sensitivity [8], but it has subsequently been found to have potent anti-inflammatory [9], [10], [11] and antioxidant [11], [12], [13] properties as well as the ability to bring about mitochondrial biogenesis [14] and to cross the blood brain barrier (BBB) [15], [16]. Metformin therapy has been successfully tested in mouse models of both Huntington's disease (OMIM 143100) and multiple sclerosis (OMIM 126200), in which, like in ALS, inflammation, oxidative stress and mitochondrial defects are thought to be contributing factors to pathology [17], [18] Specifically, oral administration of metformin has been shown to increase lifespan and improve motor performance in male mice in the R6/2 model of Huntington's disease [16] and to attenuate pathology and inhibit the expression of pro-inflammatory mediators in the murine Experimental Autoimmune Encephalomyelitis (EAE) model of multiple sclerosis [19]. In light of the observed beneficial effects of metformin therapy in these disease models of neurodegeneration, we hypothesised that metformin therapy would also attenuate motor neuron degeneration in the SOD1^G93A^ mouse model of ALS [20].

We performed initial investigations in which chronic treatment with 2 mg/ml metformin in the drinking water from 35 days of age, led to decreased motor unit loss in the hindlimbs of both male and female SOD1^G93A^ mice at 100 days of age (an early symptomatic time point [21]) Consequently, we performed a blinded, high powered, dose response survival study, using an optimised study format developed by the ALS Therapy Development Institute (ALSTDI) [22], to assess the effect of chronic metformin treatment on disease onset, progression and survival in SOD1^G93A^ mice. Surprisingly, metformin had no beneficial effects in male or female mice and furthermore, had a negative effect on the onset of neurological symptoms and disease progression in female mice.

## Materials and Methods

### Ethics Statement

All animal experiments were carried out under license from the Home Office (UK) in accordance with The Animals (Scientific Procedures) Act 1986 and were approved by Imperial College/Royal Veterinary College ethical committees (note: neither body provides an approval number).

### Mouse breeding and maintenance

For all experiments, mice were housed in a minimal disease facility, with a 12∶12 hour light∶ dark cycle and food and either water or metformin solution ad libitum. Male mice overexpressing human SOD1 with the ALS-causing G93A mutation [B6SJL-TgN SOD1 ^G93A^) 1 Gur/J, Stock number 002726] [20] were originally purchased from the Jackson Laboratory (Bar Harbor, ME). They were subsequently maintained (for over 25 generations), as hemizygotes through breeding with wild-type F1 hybrid C57Bl/6×CBA/Ca females. Mice on this background [23] have similar lifespans [24], [25] and disease progression characteristics (as measured by hindlimb electrophysiological parameters) [23], [26], to mice on the original B6SJL background and no changes in disease progression or lifespan have been seen with successive generations in our laboratory.

### Genotyping and copy number assessment

SOD1^G93A^ mice were genotyped by PCR from an ear biopsy using the following primers: forward: 5′-CATCAGCCCTAATCCATCTGA-3′ and reverse: 5′-CGCGACTAACAATCAAAGTGA-3′. Following death, a tail tip was taken from all experimental mice for potential copy number assessment via real time quantitative PCR (Q-PCR) as previously described by Alexander et al., [27]. Copy number assessment was performed for all mice used for motor unit quantification and any mice showing an outlying disease time course in our survival study. Mice showing copy number loss were excluded. Genotyping and copy number assessment protocols are described in detail in the supporting information (Method S1).

### Motor unit quantification study

#### Metformin treatment

Male and female SOD1^G93A^ mice were supplied with either normal drinking water or a solution of 2 mg/ml metformin in the drinking water from 35 days of age. Solutions were changed weekly. At 100 days of age (+/−2 days), the number of functional motor units present in the right Tibialis Anterior (TA) and Extensor Digitorum Longus (EDL) muscles of both treated and control mice was analysed via *in vivo* electrophysiology as described below. A minimum of 6 mice per sex were used in both the control and treatment groups in this study.

#### Motor unit assessment via in vivo electrophysiology

This method is routinely performed in our laboratory and has been previously described [23], [28]. Mice were deeply anesthetized via intra peritoneal injection of fentanyl/fluanisone (Hypnorm, Vetapharma Ltd.), midazolam (Hypnovel, Roche) and water (1∶1∶2 by volume) at a dosage of 9 ml/kg. Careful monitoring of mice was performed throughout the experiment and additional doses of anaesthetic were administered as required to ensure that there was no reflex response to toe pinch. The distal tendons of the right TA and EDL muscles were dissected free from surrounding tissue, individually tied with 4.0 braided surgical silk (Interfocus) and cut at their distal ends. The sciatic nerve was then exposed and all its branches cut except the common peroneal nerve (CPN), which innervates the TA and EDL muscles. The mouse was placed on a thermopad (Harvard Apparatus) to maintain body temperature at 37°C. The right foot was secured to a platform and the knee immobilized using a stainless steel pin. The TA and EDL muscle tendons were then individually attached to a strain gauge (Dynamometer, UFI Devices) to record muscle twitch forces. Isometric twitch contractions were elicited by stimulating the distal part of the DPN via bipolar silver electrodes using square wave pulses of 0.02 ms. Force output from the strain gauge was recorded using an analogue to digital converter interfaced with a computer running the appropriate software (Cambridge Electronics Design Ltd). The stimulation voltage and subsequently the length of the muscle were adjusted to produce the maximum isometric twitch force. The stimulus amplitude was then set to 0 V and manually increased over a range of 10 V, which resulted in discrete increments in twitch force due to the successive recruitment of motor units. With each increment in force, the stimulus amplitude was held at constant voltage for at least 5 stimuli and then increased manually until a larger twitch was generated. This procedure was repeated until there was no further increase in force, indicating that all motor units were recruited. The number of step-wise increments of force was counted for both control and metformin-treated mice in a blinded fashion and taken as an estimate of the number of motor units in the TA and EDL muscles.

#### Statistical analyses

After testing the data for normality, the number of surviving motor units in the TA and EDL muscles respectively of treated vs. non-treated male mice and treated vs. non-treated female mice were compared using individual unpaired t-tests.

### Dose-response survival study

#### Experimental groups

Twenty breeding trios each consisting of one SOD1^G93A^ transgenic male and two F1 hybrid C57B110×CBA/Ca females were set up simultaneously to generate a large cohort of 286 age matched pups to provide SOD1^G93A^ mice for enrolment in the survival study. Females were separated when pregnant to allow tracking of the lineage of resultant litters. Following genotyping and when the resultant male and female SOD1^G93A^ pups reached 35 days of age (+/−2 days), they were separated into four groups which were balanced with respect to age (+/−2 days), gender (14 males and 15 females per group) and body weight within gender (mean starting weights for all groups were within 0.5 g of each other). In order to ensure consistency in genetic background across the groups, transgenic mice from each litter were distributed evenly between the four groups in a gender specific fashion. Mice were housed within their groups in cages of either 4 or 5 mice for males and 5 mice for females.

#### Treatments

Mice were chronically treated with 0.5, 2 or 5 mg/ml metformin in the drinking water from 35 days of age. Metformin solutions were freshly prepared in sterilised bottles and changed twice weekly. Control mice received normal drinking water throughout the experiment. The identities of control and treatment groups were blinded to animal technicians and the investigator and were only revealed once all data analysis had been completed. When mice reached 100 days of age and began to show neurological symptoms, cages were fitted with long sipper tubes (Techniplast, UK) to allow easy access to water/drug solution and mice were provided daily with a standard amount of mashed diet prepared with the relevant water/drug solution. Dry food pellets were left on the bedding in addition to in the food hoppers throughout the experiment and cage conditions including bedding and enrichment were kept consistent across groups.

#### Analysis of disease progression and survival

Neurological scores for both hind limbs were assessed twice weekly for each mouse from 41 days of age. Neurological scores were assigned using a scale of 0–4 developed by the ALSTDI through detailed observations of SOD1^G93A^ mouse pathology [22], [29], [30]. Criteria used to assign each score level were:

0 - Full extension of hind legs away from lateral midline when mouse is suspended by its tail and mouse can hold this for 2 seconds, suspended 2–3 times.

1 - Collapse or partial collapse of leg extension towards lateral midline (weakness) or trembling of hind legs during tail suspension.

2 - Toes curl under at least twice during walking of 12 inches or any part of foot is dragging along cage bottom (a clean cage containing no bedding was used for this analysis to permit easy viewing of the toes).

3 - Rigid paralysis or minimal joint movement, foot not being used for forward motion.

4 - Mouse cannot right itself within 30 s from one or both of its sides (this test was performed in a clean cage containing the same type and amount of bedding as present in the mouse's home cage).

Body weight for all mice was measured twice weekly from 35 days of age. This provided an additional, non-invasive, measure of disease onset and progression and also allowed monitoring of the general health of the mice. Weight-loss in high copy SOD1^G93A^ mice is normally a gradual process that begins at approximately 90 days of age and continues until death. Rapid losses of weight over a short number of days that do not fit the normal pattern can therefore be indicative of non-ALS related or drug-induced maladies [22].

For humane reasons, a uniform end point of the inability of a mouse to right itself within 30 s of being placed on a side (neurological score 4) was employed for all mice. From 100 days of age, the righting reflex of each mouse from each side was tested twice daily to ensure that the end point of each mouse was accurately determined. Once mice reached their end point, they were euthanized via cervical dislocation and the date and cause of their death was recorded.

#### Graphical representation of data and statistical analyses

As the time course of pathology in male and female SOD1^G93A^ mice has been shown to be different [31] and administered therapeutics may behave differently in male and female systems, all graphical representations and statistical analyses were performed separately for males and females. In order visualise overall changes in body weight and neurological score, mean body weight and mean neurological score were plotted for each group over time until the time point at which the last mouse in the group died. To prevent decomposition of mean weight and neurological score values as mice reached their end stage, final weight and neurological score values for individual mice were carried forward until the time point at which the last mouse in their group died. This is a practice routinely used when performing survival analyses with longitudinal monitoring of weight and neurological score [29], [30]. In order to avoid the loss of statistical validity that can occur when performing repeated analyses on correlated serial measurements for the same subjects, we selected relevant summary measures for weight and neurological score data as advised by Matthews, et al. [32] and performed statistical analyses on these. Summary measures selected were time to attain peak weight (the earliest indicator of disease onset), time from peak weight to death (an indicator of disease progression), time to attain a neurological score of 2 in both hind limbs (defined as the definitive onset of symptomatic neurological disease) and finally time to reach a score of 4 (the uniform end point selected for this study, which was taken to represent survival). These summary measures all represent ‘time to event measures’ and were analyzed using Kaplan Meier survival fit analyses and the Logrank test for statistical significance. This test investigates the null hypothesis that the Kaplan Meier curves for all groups are identical (i.e. that the treatment did not change the time taken to reach the event being analysed). Low P-values are therefore indicative of differences between curves that did not occur due to chance. As multiple groups were being compared and they could be arranged in order of increasing metformin dose (from 0 mg/ml metformin in the control group to 0.5 mg/ml then 2 mg/ml then 5 mg/ml), we were also able to compare groups using the log rank test for trend. This test generates a P-value for the null hypothesis that there is no linear trend between group order and median time to event. Low P-values are therefore indicative of a significant trend between increasing metformin dose and the event being analysed. The threshold for significance for all analyses was set at P<0.05. In order to establish the age at which each mouse reached its peak weight, successive weight values for each mouse were subjected to spline smoothing and the time at which the maximum of the weight curve occurred was recorded. If the smoothed body weight curve for an individual mouse had multiple equal peaks, the time point at which the final peak occurred was taken. Spline smoothing of weight curves as well as all Kaplan Meier survival analyses were carried out using Graphpad Prism 5 software.

## Results

### Effect of chronic metformin treatment on motor unit number at 100 days of age

**Figure 1:**
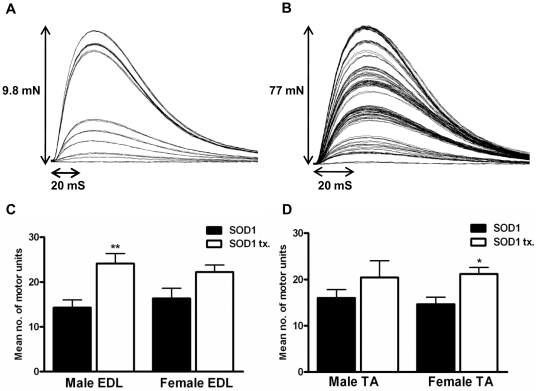
Effect of metformin treatment on motor unit number in male and female SOD1^G93A^ mice. Representative traces for *in vivo* motor unit assessment protocol (A) untreated SOD1^G93A^ male Extensor Digitorum Longus (EDL) muscle and (B) metformin treated SOD1^G93A^ EDL muscle at 100 days of age. (C) Graph showing mean number of motor units surviving in the EDL muscles of control (SOD1) and metformin treated (SOD1 tx.) SOD1^G93A^ mice at 100 days of age (SOD1^G93A^ male vs. metformin treated SOD1^G93A^ male, P = 0.005 (**)), (SOD1^G93A^ female vs. metformin-treated SOD1^G93A^ female, P = 0.056) (D) graph showing mean number of motor units surviving in the Tibialis Anterior (TA) muscles of control (SOD1) and metformin treated (SOD1 tx.) SOD1^G93A^ mice at 100 days of age (SOD1^G93A^ female vs. metformin-treated SOD1^G93A^ female, P = 0.0134 (*)). Error bars in (C) and (D) represent standard error of the mean (SEM). n = 7 SOD1 males, n = 6 SOD1 females, n = 9 metformin treated SOD1 males and n = 15 metformin treated SOD1 females.

In order to investigate the potential of metformin treatment for the attenuation of pathology in SOD1^G93A^ mice, we examined the effect of treatment of male and female SOD1^G93A^ mice with 2 mg/ml metformin in the drinking water (from 35 days of age) on motor unit survival in the right TA and EDL muscles at 100 days of age. The dose of 2 mg/ml metformin was selected as it had previously been shown to be successful in male mice in a model of Huntington's disease [16]. Motor unit survival in the muscles analysed was established by stimulating the right sciatic nerve of terminally anesthetised mice with pulses of increasing intensity and counting the number of resultant stepwise increments in twitch tension, which represented the successive recruitment of motor axons. Representative traces from these assessments are shown in Figure 1A and 1B. In these experiments, we observed a small but significant increase in the survival of functional motor units in the EDL muscles of metformin treated male SOD1^G93A^ mice compared to their untreated counterparts (24+/−2 vs. 14+/−2 motor units respectively, p<0.005) (Figure 1C) and a trend towards increased motor unit survival in the EDL muscles of metformin treated female SOD1^G93A^ mice compared to their untreated counterparts (22+/−2 vs. 16+/−2 motor units respectively, P = 0.056) (Fig. 1C). A small but significant increase in motor unit survival was also seen in the TA muscles of metformin-treated female SOD1^G93A^ mice compared to untreated female SOD1^G93A^ mice (21+/−1 vs. 15+/−2 motor units respectively, P = 0.0134) (Fig. 1D) and although there was a strong trend towards increased motor unit survival in the TA muscles of metformin-treated male SOD1^G93A^ mice compared to their untreated counterparts (20+/−4 vs. 16+/−2 motor units respectively), this did not reach significance (Fig. 1D). As a significant beneficial effect was seen in both male and female mice, we decided to further test the therapeutic potential of metformin in SOD1^G93A^ mice by performing a dose-response survival study.

### Dose response survival study

#### Metformin treatment had no effect on disease onset, progression or survival in male mice

Four groups of 14 male mice that were balanced for age, litter and body weight were enrolled in this study. The average starting bodyweights for each group were within 0.5 g of each other (Control: 20.7 g, 0.5 mg/ml: 20.5 g, 2 mg/ml: 20.2 g and 5 mg/ml: 20.6 g). During the study one male from the control group and one male from the 0.5 mg/ml group were found dead prior to developing any neurological symptoms. Additionally, one male from the 0.5 mg/ml group developed a kidney cyst and was therefore culled prematurely. These mice were all recorded as dying from ‘non ALS deaths’ and were treated as ‘censored’ in Kaplan Meier analyses and excluded from weight and neurological score plots. Two males from the 2 mg/ml group sustained fighting wounds prior to reaching end stage and were culled for humane reasons. Weight and neurological score data from these mice were included up to the date at which they were culled in weight and neurological score plots and they were treated as censored in Kaplan Meier analyses that required information about end points.

**Figure 2:**
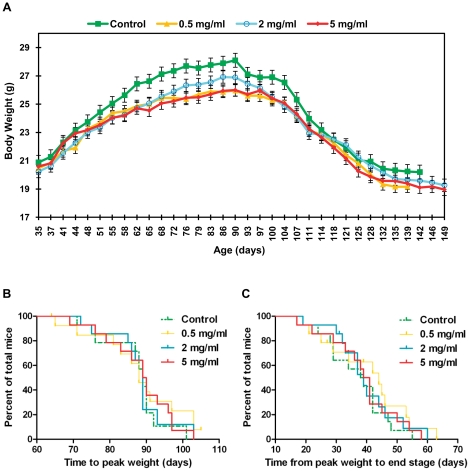
Effect of metformin on body weight, disease onset and disease progression in male SOD1^G93A^ mice. (A) Mean group body weight plotted for male SOD1^G93A^ mice over time from 35 days of age until the date at which the last death within each individual group occurred. As mice within groups died at different ages, the final weights of all mice were carried forward when calculating group mean values, until the date at which the last mouse within each group died. This prevented decomposition of the mean at later stages. Error bars represent standard error of the mean (SEM). (B) Kaplan-Meier survival plot for time to peak weight (an indicator of disease onset) in male SOD1^G93A^ mice. (C) Kaplan-Meier survival plot for time from peak weight to end stage (an indicator of disease progression) in male SOD1^G93A^ mice. Graphs (A), (B) and (C) present data for male SOD1^G93A^ mice in all four experimental groups: Control = green, 0.5 mg/ml metformin = yellow, 2 mg/ml metformin = blue, 5 mg/ml metformin = red). Results for statistical analyses performed on data presented in (B) and (C) are given in Table 1.

High copy SOD1^G93A^ mice, unlike wild type (WT) mice are unable to maintain body weight from approximately 90 days of age and steadily lose weight from this time point until death. Longitudinal monitoring of weight therefore provides a good indication of disease onset and progression and can reveal important information about the efficacy of potential therapeutics [22]. The change in mean body weight over time of male mice in all four groups is plotted in Figure 2A from 35 days onwards. Weight curves for male mice in all groups showed a very similar shape with the onset of weight decline, occurring at ∼90 days in each group. Consequently, no significant differences were observed in the selected summary measures of time to peak weight (Figure 2B, Table 1) and time from peak weight to end stage (Figure 2C, Table 1). However mice in all treatment groups appeared to be lighter throughout the study than control mice.

**Table 1:** Kaplan Meier time to event analyses for male SOD1^G93A^ mice.^1^^,^ 2, 3

	Median Value (days)				P Value	
	Control	0.5 mg/ml	2 mg/ml	5 mg/ml	Log- rank	Log-rank test for trend
**Time to peak weight**	89	88	89	89.5	0.8469	0.8492
**Time from peak weight to end stage**	38.5	44	39	40	0.6986	0.8963
**Time to reach score 2 in both hindlimbs**	114	121	114.5	114	0.6942	0.6289
**Survival**	123	128	126	126	0.8575	0.6597

1 The Logrank test investigates the null hypothesis that that the Kaplan Meier curves for all groups are identical (i.e. that the treatment did not change the time taken to reach the event being analysed). Low P-values are therefore indicative of differences between curves that did not occur due to chance. The threshold for significance was set at P<0.05.

2 To perform the log-rank test for trend, experimental groups were arranged in order of increasing dose (Control, then 0,5 mg/ml, 2 mg/ml and 5 mg/ml) and a P value testing the null hypothesis that there was no linear trend between group order and median time to event was then calculated. Low P-values are indicative of a significant trend between increasing dose and the event being analysed. The threshold for significance was set at P<0.05.

3 The time at which peak body weight occurred for each mouse was analysed after spline smoothing of each animal's body weight values over time (using Prism 5 software) from the start of the study to the time at which the mouse died. If the smoothed body weight curve for an individual mouse had multiple equal peaks, the time point at which the final peak occurred was taken.

**Figure 3:**
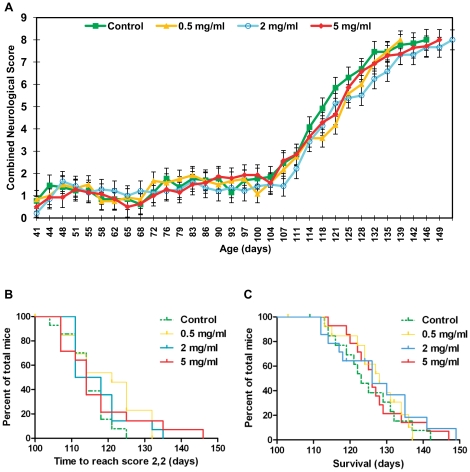
Effect of metformin on neurological score, neurological disease onset and survival in male SOD1^G93A^ mice. (A) Mean group combined neurological score plotted for male SOD1^G93A^ mice over time from 41 days of age until the date at which the last death within each group occurred. Combined neurological score was calculated for each mouse at each time point through summation of the neurological scores recorded for its left and right hindlimbs. As mice within groups died at different ages, the final combined neurological scores of all mice were carried forward when calculating group mean values, until the date at which the last mouse within each group died. This prevented decomposition of the mean at later stages. Error bars represent standard error of the mean (SEM). (B) Kaplan-Meier survival plot for the time taken for male SOD1^G93A^ mice to reach a neurological score of 2 in both hindlimbs (an indicator of the onset of symptomatic neurological disease). (C) Kaplan-Meier survival plot for the time taken for male SOD1^G93A^ mice to reach the humane end stage of attaining a neurological score of 4 (representative of survival). Graphs (A), (B) and (C) present data for male SOD1^G93A^ mice in all four experimental groups: Control = green, 0.5 mg/ml metformin = yellow, 2 mg/ml metformin = blue, 5 mg/ml metformin = red). Results for statistical analyses performed on data presented in (B) and (C) are given in Table 1.

The ALSTDI neurological scoring system was designed to allow unbiased assessment by the investigator of disease onset, progression and severity of paralysis in SOD1^G93A^ mice [22], [29]. We found this scoring system easy to implement and each score to be clearly distinct from all others, therefore negating user bias. The change in the mean combined neurological score (the sum of the neurological score recorded for left and right hind limbs) for male mice in all four groups from 41 days of age, is plotted in Figure 3A. Neurological score curves for all male groups were largely similar in shape. Specifically, scores remained low and fairly constant until approximately 104 days of age when they began to sharply increase, indicating the onset of neurological symptoms. This timepoint is approximately 14 days later than the onset of weight loss shown in Figure 2A, indicating that weight loss precedes overt neurological pathology in our SOD1^G93A^ mice. As has previously been described [29], [30], we classified the definitive onset of symptomatic neurological disease as the time point at which a mouse reached a neurological score of two in both hindlimbs. Comparison of the timepoint at which this occurred in all four males groups revealed no statistically significant differences (Figure 3B, Table 1).

For humane reasons, a uniform end point of the inability of a mouse to right itself within 30 s of being placed on a side was used for all mice in this study. The proportion of male mice surviving over time is shown in (Figure 3C). There were no statistically significant differences in survival between any groups indicating that metformin had no effect on survival in male mice (Table 1).

#### Metformin treatment negatively affected disease onset and progression in female mice in a dose-dependent fashion

Four groups of 15 female mice that were balanced for age, litter and bodyweight were enrolled in this study. The average starting bodyweights for all groups were within 0.5 g of each other (Control: 16.7 g, 0.5 mg/ml: 16.9 g, 2 mg/ml: 16.8 g and 5 mg/ml: 17.2 g). Towards the end of the study, one female mouse from the 0.5 mg/ml group was injured by a fellow cage mate and was therefore culled. Weight and neurological score data from this mouse were included in weight and neurological score plots up to the date at which she became wounded and she was treated as censored in any Kaplan Meier analyses requiring information about survival. One further female mouse from the 0.5 mg/ml group showed abnormally rapid weight loss and signs of discomfort over the course of two days without showing neurological symptoms. She was therefore culled and classed as dying a ‘non-ALS death’. Weight and neurological score data from this mouse were not included weight and neurological score plots and she was treated as censored in all Kaplan Meier analyses.

**Figure 4:**
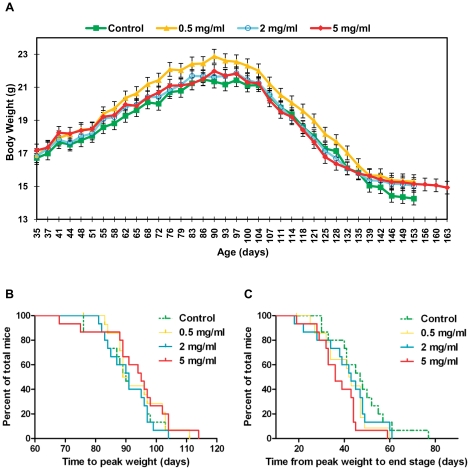
Effect of metformin on body weight, disease onset and disease progression in female SOD1^G93A^ mice. (A) Mean group body weight plotted for female SOD1^G93A^ mice over time from 35 days of age until the date at which the last death within each individual group occurred. As mice within groups died at different ages, the final weights of all mice were carried forward when calculating group mean values, until the date at which the last mouse within each group died. This prevented decomposition of the mean at later stages. Error bars represent the standard error of the mean (SEM). (B) Kaplan-Meier survival plot for time to peak weight (an indicator of disease onset) in female SOD1^G93A^ mice. (C) Kaplan-Meier survival plot for time from peak weight to end stage (an indicator of disease progression) in female SOD1^G93A^ mice. Graphs (A), (B) and (C) present data for female SOD1^G93A^ mice in all four experimental groups: Control = green, 0.5 mg/ml metformin = yellow, 2 mg/ml metformin = blue, 5 mg/ml metformin = red). Results for statistical analyses performed on data presented in (B) and (C) are given in Table 2.

The change in mean body weight over time for female mice in all four groups is plotted in Figure 4A from 35 days onwards. As with male mice, weight curves for all groups showed a very similar shape, with the onset of weight decline occurring at approximately 90 days of age. However, female mice in the 0.5 mg/ml group tended to be heavier than mice in other groups between 55 and 135 days of age and female mice in the control group tended to drop to lower weights at end stage than mice in treatment groups. As with male mice, no significant differences were seen between female groups in the time taken to reach peak weight (Figure 4B, Table 2) but unlike male mice, there was a significant association between increasing metformin dose and decreasing time from peak weight to end stage in female mice (Figure 4C, Table 2, P = 0.0362). This indicates that metformin had a negative effect on disease progression in female mice.

**Table 2:** Kaplan Meier time to event analyses for female SOD1^G93A^ mice^1^^,^ 2 ^,^ 3.

	Median Value (days)				P Value	
	Control	O.5 mg/ml	2 mg/ml	5 mg/ml	Log- rank	Log-rank test for trend
**Time to peak weight**	90	90	91	95	0.5418	0.3664
**Time from peak weight to end stage**	47	42.5	43	36	0.1149	0.0362 Significant
**Time to reach score 2 in both hindlimbs**	132	125	118	118	0.0991	0.0236 Significant
**Survival**	140	136	132	132	0.6216	0.1964

1 The Logrank test investigates the null hypothesis that that the Kaplan Meier curves for all groups are identical (i.e. that the treatment did not change the time taken to reach the event being analysed). Low P-values are therefore indicative of differences between curves that did not occur due to chance. The threshold for significance was set at P<0.05.

2 To perform the log-rank test for trend, experimental groups were arranged in order of increasing dose (Control, then 0,5 mg/ml, 2 mg/ml and 5 mg/ml) and a P value testing the null hypothesis that there was no linear trend between group order and median time to event was then calculated. Low P-values are indicative of a significant trend between increasing dose and the event being analysed. The threshold for significance was set at P<0.05.

3 The time at which peak body weight occurred for each mouse was analysed after spline smoothing of each animal's body weight values over time (using Prism 5 software) from the start of the study to the time at which the mouse died. If the smoothed body weight curve for an individual mouse had multiple equal peaks, the time point at which the final peak occurred was taken.

**Figure 5:**
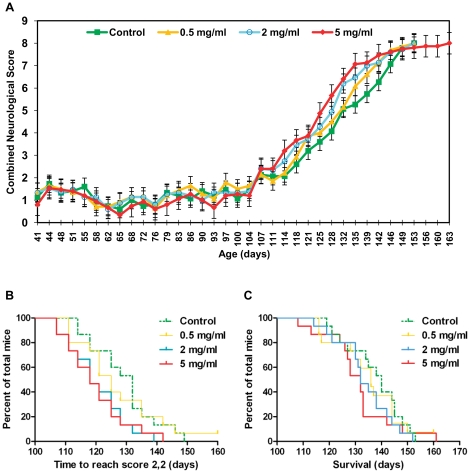
Effect of metformin on neurological score, neurological disease onset and survival in female SOD1^G93A^ mice. (A) Mean group combined neurological score plotted for female SOD1^G93A^ mice over time from 41 days of age until the date at which the last death within each group occurred. Combined neurological score was calculated for each mouse at each time point through summation of the neurological scores recorded for its left and right hindlimbs. As mice within groups died at different ages, the final combined neurological scores of all mice were carried forward when calculating group mean values, until the date at which the last mouse within each group died. This prevented decomposition of the mean at later stages. Error bars represent standard error of the mean (SEM). (B) Kaplan-Meier survival plot for the time taken for female SOD1^G93A^ mice to reach a neurological score of 2 in both hindlimbs (an indicator of the onset of symptomatic neurological disease). (C) Kaplan-Meier survival plot for the time taken for female SOD1^G93A^ mice to reach the humane end stage of attaining a neurological score of 4 (representative of survival). Graphs (A), (B) and (C) present data for female SOD1^G93A^ mice in all four experimental groups: Control = green, 0.5 mg/ml metformin = yellow, 2 mg/ml metformin = blue, 5 mg/ml metformin = red). Results for statistical analyses performed on data presented in (B) and (C) are given in Table 2.

The change in the mean combined neurological score (the sum of the neurological score recorded for left and right hind limbs) for female mice in all four groups from 41 days of age, is plotted in Figure 5A. Neurological score curves for female mice in all groups were similar in shape until approximately 104 days of age when, as with male mice, the onset of neurological symptoms began. Following this point, however, neurological score curves began to rise more steeply with increasing metformin dose, suggesting a negative effect of metformin on disease progression. In support of this there was a significant association between increasing metformin dose and the time point at which female mice reached a score of 2 in both hindlimbs (the definitive onset of symptomatic disease) (Figure 5B, Table 2, P = 0.0236).

The proportion of female mice surviving over time is shown in (Figure 5C). Although there were no significant differences in survival between groups (Table 2), there was a trend towards increasing survival with decreasing metformin dose.

## Discussion

ALS is a devastating neurodegenerative disorder that is in desperate need of suitable treatments. Consequently, FDA-approved drugs that are routinely used for the treatment of other diseases, represent attractive candidates for research as potential ALS therapeutics, as their previous safety, toxicity and pharmacological testing would speed up their passage into clinical trial [33]. Metformin is a routinely used anti-type II diabetes drug, which has been shown to have anti-inflammatory and antioxidant properties as well as the ability to bring about mitochondrial biogenesis. In recent years, metformin has been shown to attenuate pathology in mouse models of both Huntington's disease [16] and multiple sclerosis [19] which, like ALS models, show central nervous system (CNS)-based inflammation, oxidative stress and mitochondrial abnormalities [4], [17], [18]. We therefore hypothesised that metformin would also attenuate pathology in the SOD1^G93A^ mouse model of ALS. We tested this hypothesis through an initial study involving the measurement of motor unit number in the TA and EDL muscles of treated mice at the symptomatic timepoint of 100 days of age and then performed a dose-response survival study with longitudinal tracking of disease progression to further assess the potential of metformin for ALS therapy.

The ultimate cause of death in SOD1^G93A^ mice is the retraction of distal motor axons and denervation of skeletal muscles [34], [35]. In light of this, bringing about the preservation of functional motor units, in which the motor neuron is not only present but also forming functional neuromuscular junctions, is likely to be critical when trying to reduce the severity of disease pathology in these animals. We therefore reasoned that analysis of the effect of metformin treatment from 35 days of age, on functional motor unit survival at the symptomatic time point of 100 days of age, would provide a good indication of the potential neuroprotective properties of this drug. In these investigations we observed a significant increase in the survival of functional motor units as measured by *in vivo* electrophysiology, in metformin-treated male EDL muscles (24+/−2 vs. 14+/−2 motor units, p<0.005) as well as in metformin-treated female TA muscles (21+/−1 vs. 15+/−2 motor units, P = 0.0134). A strong trend towards increased motor unit survival was also observed in female EDL muscles and male TA muscles. We have previously determined the number of functional motor units in wild type (WT) male EDL muscles at 100 days of age to be 54+/−4 (n = 6). The increase in the survival of functional motor units seen in the EDL muscles of metformin-treated male mice therefore represented a moderate increase from approximately 26% of WT motor units to 44%. The number of functional motor units in the TA muscles of WT mice has been determined by Hegedus et al., to be 95+/−12.4 [36]. The increase in survival of functional motor units seen in the TA muscles of metformin-treated female mice therefore represented a smaller increase from approximately 16% of WT motor units to 22%. Although these increases in motor unit survival seen were not sufficient to restore motor unit numbers to levels comparable to WT, they suggested that metformin was having a trophic effect on motor neuron health in SOD1^G93A^ mice at 100 days of age and highlighted the possibility that motor unit survival may be further enhanced with a different dose of metformin. This led us to perform a dose response, survival study to investigate the effects of metformin on disease onset, progression and survival in these mice.

Despite successful results being documented for many therapeutics tested in the SOD1^G93A^ mouse, Riluzole has been the only drug to show any, albeit small, beneficial effect in ALS patients [37]. Investigations into the potential reasons for this disparity have revealed several confounding variables that may lead to false positives in experiments in SOD1^G93A^ mice [22]. These variables include, in order of impact: the occurrence of non-ALS deaths, the incidence of low copy transgenics, genetic background or epigenetic influences causing littermate clustering and a slight gender effect [22]. Identification of these variables has lead to the production of guidelines for the preclinical testing of therapeutics for ALS, which aim to control for these variables and increase study validity [22], [38]. We carefully followed these guidelines in order to ensure as far as possible, the accurate determination of the potential of metformin as a therapy for ALS. Specifically in order to control for the variability introduced by non-ALS deaths, we closely monitored the health of all mice throughout the study and excluded relevant data from any mice observed to experience a non-ALS death. We checked the copy number of any mice displaying outlying disease progression or survival characteristics by Q-PCR [27] and were prepared to exclude any mice which had lost copies of the transgene (although no mice with fewer copies were identified in this study). Experiments in this study were performed with mice on a mixed-hybrid genetic background. Mice on backgrounds such as this show a greater variability than those from inbred strains [39] and therefore it was essential that we controlled for the effects of litter clustering in order to produce meaningful results. Scott et al., confirmed using a specially designed computer model (SimLIMS) and data from 2241 control SOD1^G93A^ mice on a mixed hybrid genetic background (maintained by breeding hemizygous B6SJLTg (SOD1^G93A^) males to B6SJLF1 dams), that with an n-number of 24 mice per cohort (12 males and 12 females), and the employment of same-gender litter matching as well as exclusion of mice experiencing non-ALS deaths or loss of transgene copies, noise within the experiment could be reduced to a virtual zero [22]. We were careful to meet these requirements and are therefore confident that our results are meaningful.

Despite seeing small but significant increases in motor unit survival with metformin treatment in our initial investigations, we were unable to detect any significant effect of metformin treatment on disease onset, progression or survival in male or female SOD1^G93A^ mice at any of the doses tested in our survival study. This suggests that at the early symptomatic timepoint of 100 days of age, when motor unit survival was measured in our initial study, metformin-induced beneficial effects outweighed any toxic effects and were therefore able to augment disease pathology. However, at later time points, metformin induced trophic effects may have been overshadowed by advancing and aggressive SOD1^G93A^ pathology and potentially negative drug effects. This highlights that experiments which provide a ‘snapshot’ of pathology at a particular time point, should always be backed up with robust survival studies including longitudinal monitoring of disease progression, before drugs are put forward for clinical trial.

Metformin has previously been shown to have anti-inflammatory and anti-oxidant properties as well as the ability to bring about mitochondrial biogenesis and these effects are likely to have contributed to the increased motor unit survival seen in our initial study. However, this drug has been identified as having many other cellular effects in addition to these and some of these could have been detrimental in SOD1^G93A^ mice and prevented longterm attenuation of motor unit loss. For example, metformin has been shown to inhibit mitochondrial complex one [40], which is already compromised as a result of ALS pathology [41] and also to bring about a reduction in both total-and LDL-cholesterol levels, high levels of which have paradoxically been shown to be a positive prognostic factor in ALS patients [42].

In our survival study we were surprised to observe a significant association between increasing metformin dose and both the onset of symptomatic neurological disease and disease progression in female SOD1^G93A^ mice. This suggests that metformin may have interacted in a negative fashion with a female specific cellular process that is normally beneficial for motor neuron health. It is well documented that female SOD1^G93A^ mice develop neurological symptoms later and have a longer lifespan than male SOD1^G93A^ mice and this was also found to be the case in our study (Figure S1A and B, Tables S1 and S2). Specifically, median onset of symptomatic neurological disease was 18 days later in control female mice than in their male counterparts (132 vs. 114, P = 0.0001) and median survival was extended by 17 days (140 vs. 123, P = 0.0011). Interestingly, prevention of oestrogen production in female SOD1^G93A^ mice via ovariectomy has been shown to accelerate disease progression [31] and also to reduce survival time to levels comparable to male SOD1^G93A^ mice [31], [43]. Furthermore treatment of these ovariectomised females with 17β-oestradiol has been shown to rescue these effects [31]. This suggests that the presence of oestrogen is a large contributing factor to the slower disease progression and increased lifespan of female SOD1^G93A^ mice compared to males. Metformin is increasingly becoming prescribed for the treatment of polycystic ovary syndrome (PCOS [OMIM 184700]) with the aim of reducing the insulin resistance that is associated with the disorder. Interestingly, it has recently been identified that metformin is also able to inhibit basal and insulin-stimulated 17β-ostradiol (E2) production and that this may contribute to its beneficial effect in PCOS [44], [45]. In light of the trophic effect of oestrogen on disease progression and survival in female SOD1^G93A^ mice and the documented ability of metformin to inhibit oestrogen production, it seems likely that the negative effects of metformin seen in female mice in our study were at least in part derived from reduced oestrogen production. In support of this, treatment of female SOD1^G93A^ mice with either 2 or 5 mg/ml metformin, prevented the significant delay in the onset of symptomatic neurological disease seen in female control mice compared to male control mice (Figure S1A, Table S2). Furthermore, treatment of female SOD1^G93A^ mice with metformin at all doses negated the significant increase in survival seen in female control mice compared to male control mice and this effect became more pronounced with increasing metformin dose (Figure S1B, Table S2).

In conclusion, we have shown that despite showing promise in mouse models of Huntington's disease and multiple sclerosis, which share many pathological processes with SOD1^G93A^ mice, metformin is not capable of delaying disease onset, slowing disease progression or increasing survival in these animals. Furthermore, metformin appears to be harmful in female SOD1^G93A^ mice in a dose dependent fashion and this effect may be derived from its ability to inhibit oestrogen production. This could have implications for the prescription of medication to female ALS patients suffering from type II diabetes as metformin could potentially worsen their prognosis. However this situation is likely to be complex as a recent retrospective study of ALS patients with pre-morbid diabetes mellitus has suggested a positive effect of diabetes on age of ALS onset [46]. Further investigations into the relationship between ALS and diabetes and the way in which metformin treatment modifies this relationship are therefore required. Finally, the disparity between the observed ability of metformin to moderately increase motor unit survival in our initial investigations and the inability of this beneficial effect to be translated into delayed disease onset and progression or increased survival, highlights that studies analyzing the effect of a drug at a single time point are alone not sufficient to warrant the testing of this drug in the ALS clinic.

## Supporting Information

Figure S1 Kaplan-Meier time to event plots for (A) time taken for mice to reach a score of 2 in both hindlimbs (the definitive onset of symptomatic neurological disease) and (B) time taken for mice to reach the humane end stage of the inability to right within 30 s of being placed on a side (survival) for all female groups and the male control group. Female mice were treated with normal drinking water (control, green) or 0.5 (yellow), 2 (blue) or 5 (red) mg/ml metformin in the drinking water from 35 days of age. Male control mice (black) received normal drinking water throughout.
(TIF)

Table S1 Median values derived from Kaplan Meier analyses for the time taken for mice to reach a score of 2 in both hindlimbs and the time taken for mice to reach the humane end stage of the inability to right within 30 s of being placed on a side (survival) for all female groups (control, 0.5, 2 and 5 mg/ml metformin) and the male control group.
(DOC)

Table S2 Summary of statistical analyses performed to compare the time taken for mice to reach a score of 2 in both hindlimbs and the time taken for mice to reach the humane end stage of the inability to right within 30 s of being placed on a side in all experimental groups.The Logrank test investigates the null hypothesis that that the Kaplan Meier curves for all groups are identical. Low P-values are therefore indicative of differences between groups that did not occur due to chance. Statistical comparison of the time taken for mice to reach a score of 2 in both hindlimbs and the time taken for mice to reach the humane end stage (survival) in all four male and female groups via a log rank test revealed that there were significant differences between the groups for both measures. The threshold for significance was set at P<0.05 for these comparisons. Subsequent post hoc comparisons between pairs of groups for both measures were then performed. Using Bonferroni's correction for multiple comparisons we calculated that P must be less than 0.0018 (i.e. P = <0.05/28, where 28 represents the number of possible pairwise comparisons for 8 different experimental groups) in order to be significant in these pairwise comparisons.
(DOC)

Method S1 Genotyping and copy number assessment.
(DOC)
