# Supplementary figures and images for: Metformin Treatment Has No Beneficial Effect in a Dose-Response Survival Study in the SOD1G93A Mouse Model of ALS and Is Harmful in Female Mice
Source: PLoS One. 2011 Sep 1;6(9):e24189. doi: 10.1371/journal.pone.0024189 (PMC3164704; doi:10.1371/journal.pone.0024189)

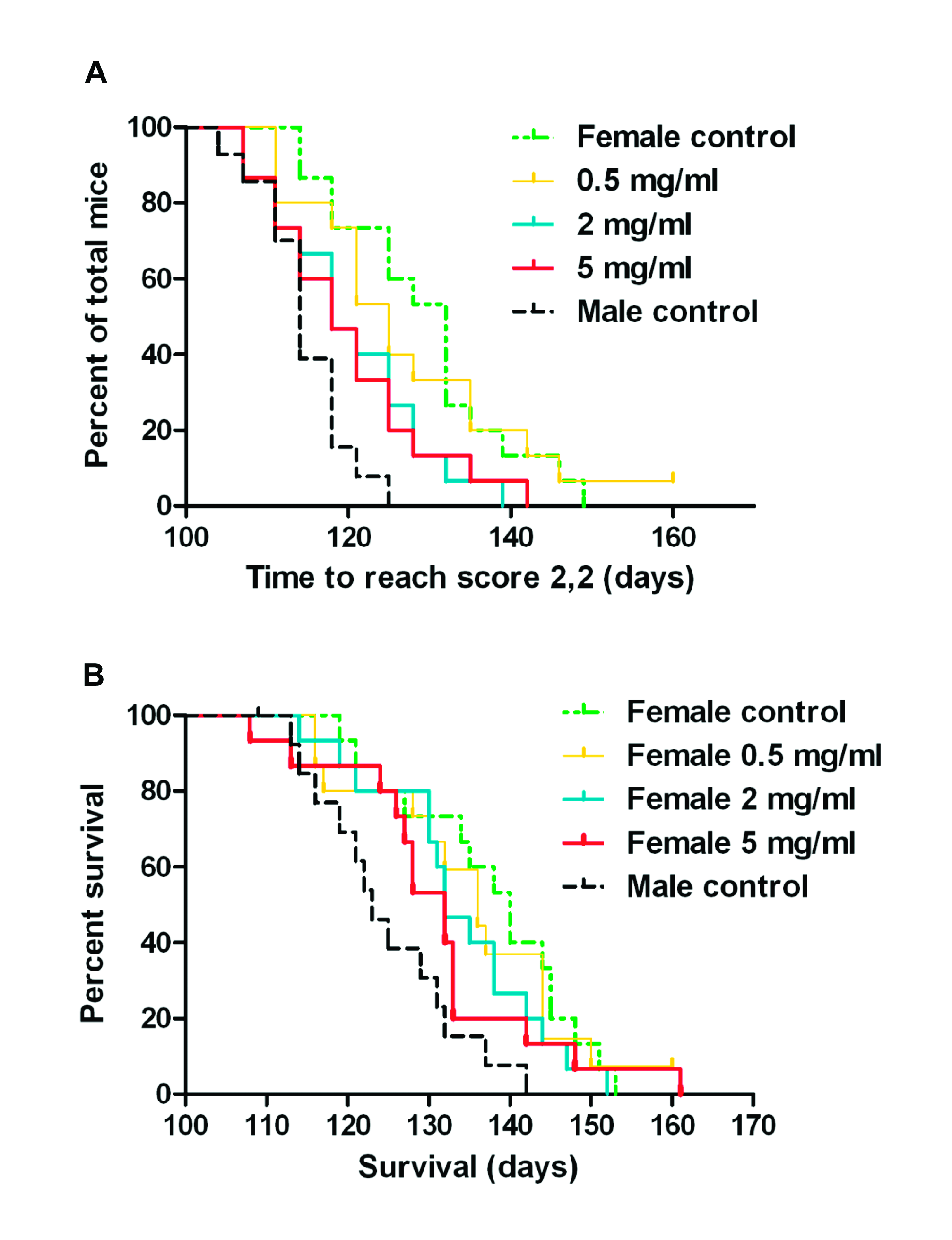

Supplement: Figure S1 — Kaplan-Meier time to event plots for (A) time taken for mice to reach a score of 2 in both hindlimbs (the definitive onset of symptomatic neurological disease) and (B) time taken for mice to reach the humane end stage of the inability to right within 30 s of being placed on a side (survival) for all female groups and the male control group. Female mice were treated with normal drinking water (control, green) or 0.5 (yellow), 2 (blue) or 5 (red) mg/ml metformin in the drinking water from 35 days of age. Male control mice (black) received normal drinking water throughout. (TIF) [file pone.0024189.s001.tif]
