# Supplementary material for: Metformin Treatment Has No Beneficial Effect in a Dose-Response Survival Study in the SOD1G93A Mouse Model of ALS and Is Harmful in Female Mice
Source: PLoS One. 2011 Sep 1;6(9):e24189. doi: 10.1371/journal.pone.0024189 (PMC3164704; doi:10.1371/journal.pone.0024189)
Supplement: Table S1 — Median values derived from Kaplan Meier analyses for the time taken for mice to reach a score of 2 in both hindlimbs and the time taken for mice to reach the humane end stage of the inability to right within 30 s of being placed on a side (survival) for all female groups (control, 0.5, 2 and 5 mg/ml metformin) and the male control group. (DOC) [file pone.0024189.s002.doc]

**Table S1**

|  | **Median Value (days)** | | | | |
| --- | --- | --- | --- | --- | --- |
|  | **Female control** | **Female 0.5mg/ml** | **Female 2mg/ml** | **Female 5mg/ml** | **Male Control** |
| **Time to reach score 2,2** | 132 | 125 | 118 | 118 | 114 |
| **Survival** | 140 | 136 | 132 | 132 | 123 |

Median values derived from Kaplan Meier analyses for the time taken for mice to reach a score of 2 in both hindlimbs and the time taken for mice to reach the humane end stage of the inability to right within 30s of being placed on a side (survival) for all female groups (control, 0.5, 2 and 5 mg/ml metformin) and the male control group.
