# Supplementary material for: Metformin Treatment Has No Beneficial Effect in a Dose-Response Survival Study in the SOD1G93A Mouse Model of ALS and Is Harmful in Female Mice
Source: PLoS One. 2011 Sep 1;6(9):e24189. doi: 10.1371/journal.pone.0024189 (PMC3164704; doi:10.1371/journal.pone.0024189)
Supplement: Table S2 — Summary of statistical analyses performed to compare the time taken for mice to reach a score of 2 in both hindlimbs and the time taken for mice to reach the humane end stage of the inability to right within 30 s of being placed on a side in all experimental groups.The Logrank test investigates the null hypothesis that that the Kaplan Meier curves for all groups are identical. Low P-values are therefore indicative of differences between groups that did not occur due to chance. Statistical comparison of the time taken for mice to reach a score of 2 in both hindlimbs and the time taken for mice to reach the humane end stage (survival) in all four male and female groups via a log rank test revealed that there were significant differences between the groups for both measures. The threshold for significance was set at P<0.05 for these comparisons. Subsequent post hoc comparisons between pairs of groups for both measures were then performed. Using Bonferroni's correction for multiple comparisons we calculated that P must be less than 0.0018 (i.e. P = <0.05/28, where 28 represents the number of possible pairwise comparisons for 8 different experimental groups) in order to be significant in these pairwise comparisons. (DOC) [file pone.0024189.s003.doc]

**Table S2**

|  | **P Value, Time to reach score 2,2, log-rank test** | **P Value, Survival, log-rank test** |
| --- | --- | --- |
| **All male and female groups** | 0.0317 Significant | 0.0190 Significant |
| **Male control vs. female control** | 0.0001 Significant | 0.0011 Significant |
| **Male control vs. female 0.5mg/ml** | 0.0014 Significant | 0.0078 |
| **Male control vs. female 2mg/ml** | 0.0416 | 0.0179 |
| **Male control vs. female 5 mg/ml** | 0.0804 | 0.0870 |

Summary of statistical analyses performed to compare the time taken for mice to reach a score of 2 in both hindlimbs and the time taken for mice to reach the humane end stage of the inability to right within 30s of being placed on a side in all experimental groups.The Logrank test investigates the null hypothesis that that the Kaplan Meier curves for all groups are identical. Low P-values are therefore indicative of differences between groups that did not occur due to chance. Statistical comparison of the time taken for mice to reach a score of 2 in both hindlimbs and the time taken for mice to reach the humane end stage (survival) in all four male and female groups via a log rank test revealed that there were significant differences between the groups for both measures. The threshold for significance was set at P<0.05 for these comparisons. Subsequent post hoc comparisons between pairs of groups for both measures were then performed. Using Bonferroni's correction for multiple comparisons we calculated that P must be less than 0.0018 (i.e. P= <0.05/28, where 28 represents the number of possible pairwise comparisons for 8 different experimental groups) in order to be significant in these pairwise comparisons.
