# Supplementary material for: Metformin Treatment Has No Beneficial Effect in a Dose-Response Survival Study in the SOD1G93A Mouse Model of ALS and Is Harmful in Female Mice
Source: PLoS One. 2011 Sep 1;6(9):e24189. doi: 10.1371/journal.pone.0024189 (PMC3164704; doi:10.1371/journal.pone.0024189)
Supplement: Method S1 — Genotyping and copy number assessment. (DOC) [file pone.0024189.s004.doc]

**Supporting information: method S1**

Genotyping and copy number assessment

SOD1^G93A^ mice were genotyped by PCR from an ear biopsy using the following primers: forward: 5’-CATCAGCCCTAATCCATCTGA-3’ and reverse: 5’-CGCGACTAACAATCAAAGTGA-3’. These primers amplify a section of exon 4 of the human SOD1 gene and were first described by Rosen et al., . Upon death, a 0.5cm tail tip was removed from each experimental mouse for use in transgene copy number assessment by real time quantitative PCR (Q-PCR) as previously described by Alexander et al., . Briefly, DNA was extracted from tail tips using DNeasy blood and tissue kits (QIAGEN) and the concentration and purity of the DNA measured using a nanodrop spectrophotometer. Primer sequences used for the human SOD1 transgene were: 5′-CATCAGCCCTAATCCATCTGA-3′ (forward) and 5′-CGCGACTAACAATCAAAGTGA-3′ (reverse) and primer sequences for the mouse interleukin 2 (mIL2) reference gene were: 5′-CTAGGCCACAGAATTGAAAGATCT-3′ (forward), 5′-GTAGGTGGAAATTCTAGCATCATCC-3′ (reverse). Brilliant® II SYBR Green QPCR Master Mix reagent (Stratagene) was used for the real-time amplification and the final concentrations of forward and reverse primers used were 0.4 µM for human SOD1 and 0.5µM for mIL2. Two ng of DNA were used per reaction. Cycling conditions used were as follows: initial activation of Sure Start® Taq polymerase at 95 °C for 10 minutes, then 40 cycles of 95 °C for 30 seconds, 60 °C for 1 minute and 72 °C for 30 seconds. Assays were performed in duplicate using either of two dectection systems; the Mx3000p Q-PCR system (Stratagene) (initial pilot study) or the Chromo4 Real Time PCR-Detector (Biorad) (survival study). A no template control was performed for both human SOD1 and mIL2 primers in each Q-PCR experiment to rule out contamination of the master mix with genomic DNA. Average cycle threshold (CT) values for human SOD1 and mIL2 were determined for each sample by the detection systems used. ΔCT values, which represent a direct index of transgene copy number, were calculated by subtracting the mean human SOD1 CT value for a particular sample from its corresponding mean mIL2 CT value. In our motor unit quantification study, copy number assessment was performed for all mice and any mice with a ∆CT value that differed by more than two standard deviations from the mean value were excluded from the study. In our survival study, copy number assessment was performed for any mice displaying outlying disease progression or survival characteristics with respect to their group and the ∆CT values obtained were compared to those obtained for a subset of 4 male and 4 female control mice displaying normal survival. Any mice with a ∆CT value that differed by more than 2 standard deviations from the mean ∆CT of this control group were to be excluded, although no mice met this criteria.

1. Rosen DR, Siddique T, Patterson D, Figlewicz DA, Sapp P, et al. (1993) Mutations in Cu/Zn superoxide dismutase gene are associated with familial amyotrophic lateral sclerosis. Nature 362: 59-62.

2. Alexander GM, Erwin KL, Byers N, Deitch JS, Augelli BJ, et al. (2004) Effect of transgene copy number on survival in the G93A SOD1 transgenic mouse model of ALS. Brain researchMolecular brain research 130: 7-15.
